# Supplementary material for: Effect of an intensive cervical traction protocol on mid-term disability and pain in patients with cervical radiculopathy: An exploratory, prospective, observational pilot study
Source: PLoS One. 2021 Aug 11;16(8):e0255998. doi: 10.1371/journal.pone.0255998 (PMC8357129; doi:10.1371/journal.pone.0255998)
Supplement: Attachment — Submitted filename: response to reviewers 20210726.docx [file pone.0255998.s001.docx]

We thank the reviewers for their positive comments on our work. We hope that this version will fully satisfy them.

Reviewer #2: All comments have been addressed

Reviewer #3: (No Response)

Reviewer #4: All comments have been addressed

We thank the reviewers 2 for their validation of our comments and our new version

Reviewer #2: (No Response)

We thank the reviewer 2 for his validation of this new version

Reviewer #3: The paper is in better shape. Congratulations to the authors for such a nice work. Yet, some modifications need to be made before publication. Kindly refer to the following aspects.

1. Conclusion needs to provide answers for each objective clearly in a sentence or two.
a. Here the conclusion does not support the findings of the paper.
b. As the study was not comparative study, such statements need to be removed.
c. The conclusion should only contain information pertaining to findings of the current paper (not other studies).

We thank reviewer 3 for these remarks. We have modified the document according to this advice. We have removed the reference to the comparison of other studies line 362-364.

2. Abstract contains inadequate information.
a. Mention the design of the study clearly.
b. Methods section is not well described.

We thank reviewer 3 for this analysis. We have added the words "prospective and open" just before “observational study” line 26. To improve clarity, we have also modified the following paragraph by adding the main objective of the study. Finally, we have rewritten the following sentence to make it clearer (line 29-32). In line with precedent comment on conclusion, we change abstract’s conclusion too (line 42-44)

3. Please DELETE INFORMATION UNRELATED TO OBJECTIVE so that the section is short and sweet. Kindly shorten this section and delete unrelated information. Kindly focus on three elements of introduction
a. What is known about the topic? (Background)
b. What is not known? (The research problem)
c. Why the study was done? (Justification)

We understand this remark less. The text has been deeply reworked during our last submission in order to answer this request. It is, in fact, extremely short by keeping only the essential useful to physicians and physiotherapists involved in these tractions. We understand that other expert actors may want an even shorter text, but it seems useful to us here to keep the remaining elements to contextualize.

4. There are too many strikethrough marks. Kindly delete the text completely which has been asked to delete. At the same time, please highlight the changes that have been made.

We sent two versions, as requested by the publisher, one with the marks, the other without, to clarify the reviewers' re-reading of the changes. We have, of course, respected this request at every stage.

5. It would be better to shorten the paper. It seems there is too much of information.

We thank reviewer 3 for this comment. We have streamlined our text to meet this request.

6. Kindly mention tables and figures in line with recommendations of journal. For example, table is not required in introduction.

We understand the comment of reviewer 3. We had quoted the table in the introduction. In accordance with the recommendations to the authors, we have therefore illustrated with the table. We have made the modification requested by reviewer 3 and the table now appears in the discussion when it is quoted a second time in the text.

Reviewer #4: Thank you for your efforts. All comments have been addressed. In this study the effect of cervical traction on neuropathic pain in the case of cervical radiculopathy was assessed.

We thank the reviewer 4 for this compliment and validation of this new version
